# The Role of Ribosomal Protein S6 Kinases in Plant Homeostasis

**DOI:** 10.3389/fmolb.2021.636560

**Published:** 2021-03-10

**Authors:** Irabonosi Obomighie, Kestutis Lapenas, Billy E. Murphy, Alexander M. C. Bowles, Ulrike Bechtold, Filippo Prischi

**Affiliations:** School of Life Sciences, University of Essex, Colchester, United Kingdom

**Keywords:** ribosomal protein S6 kinases, plant homeostasis, abiotic stress, cell signaling, stress response

## Abstract

The p70 ribosomal S6 kinase (S6K) family is a group of highly conserved kinases in eukaryotes that regulates cell growth, cell proliferation, and stress response via modulating protein synthesis and ribosomal biogenesis. S6Ks are downstream effectors of the Target of Rapamycin (TOR) pathway, which connects nutrient and energy signaling to growth and homeostasis, under normal and stress conditions. The plant S6K family includes two isoforms, S6K1 and S6K2, which, despite their high level of sequence similarity, have distinct functions and regulation mechanisms. Significant advances on the characterization of human S6Ks have occurred in the past few years, while studies on plant S6Ks are scarce. In this article, we review expression and activation of the two S6K isoforms in plants and we discuss their roles in mediating responses to stresses and developmental cues.

## Introduction

A common feature of all living organisms is the ability to detect and respond to changes in the external environment such as drought, flooding, extreme temperatures, or pathogen infections, which are major yield-limiting factors (Alcazar et al., 2006). Environmental stress conditions often result in energy deprivation due to inhibition of photosynthesis (Baena-Gonzalez and Sheen, 2008), leading to the alteration of cellular processes including the induction of rapid protective mechanism (e.g., osmolyte accumulation and antioxidants) and other metabolic reprogramming promoting stress tolerance often at the expense of plant growth (Baena-Gonzalez, 2010; Bechtold et al., 2018; Bechtold and Field, 2018). Consequently, plants have evolved tightly regulated signaling pathways, which can sense changes in the environment and elicit a response (Dobrenel et al., 2011). These pathways employ kinases that work as molecular switches able to finely tune gene expression depending on the external and internal environments, resulting in a wide range of phenotypes. Studies on *Arabidopsis thaliana* (Arabidopsis) have shown that, similarly to most eukaryotes (Kozma and Thomas, 2002; Liao et al., 2008), the p70 ribosomal S6 kinases (S6Ks) pathway coordinates cell growth, cell proliferation, and stress response via modulating protein synthesis and ribosomal biogenesis (Turck et al., 1998; Mahfouz et al., 2006; Rhoads et al., 2007). Importantly, S6Ks are the downstream effectors of the Target of Rapamycin (TOR), which is considered the master regulator of growth and metabolism able to directly or indirectly regulate transcription, translation, ribosome biogenesis, translocation of regulatory proteins, autophagy, and storage of reserve compounds (Dobrenel et al., 2011). In plants, TOR is activated in response to high nutrient availability, while under low carbon conditions Snf1-Related Protein Kinase 1 (SnRK1) is activated and, in an opposing way to TOR, promotes energy saving (Robaglia et al., 2012; Margalha et al., 2019).

**FIGURE 1:**
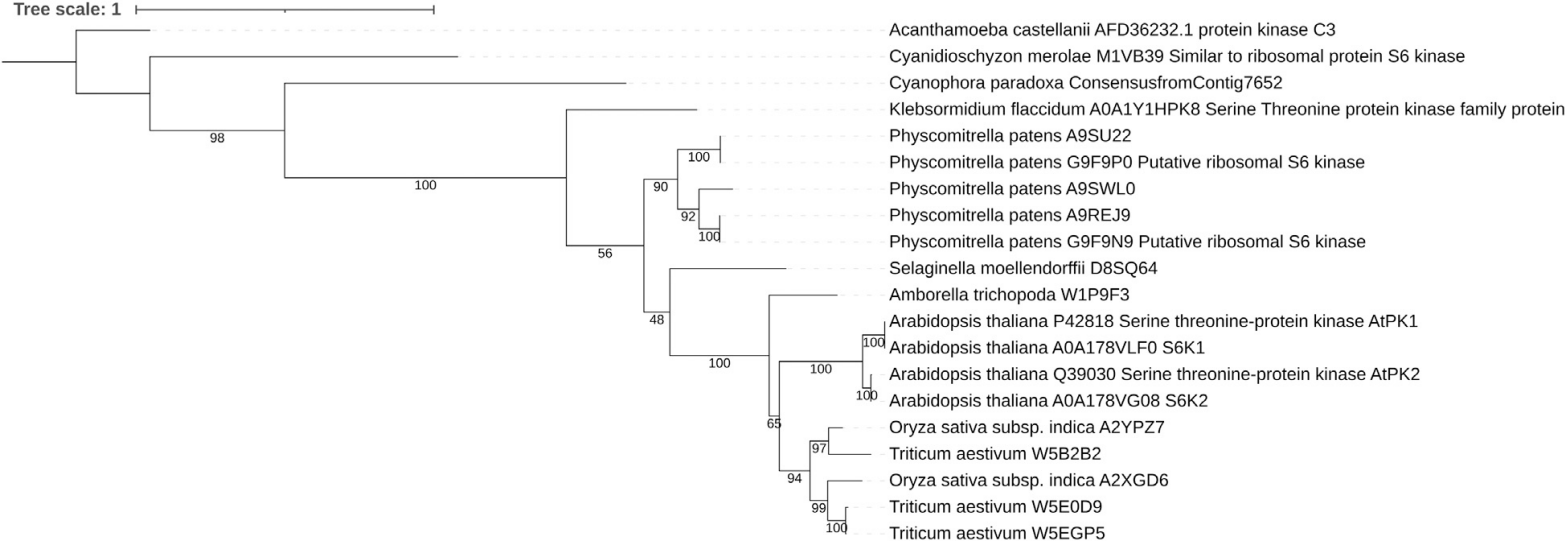
Maximum likelihood tree of plant S6K1 and S6K2. In all plant proteins the general domain structure as well as the kinase domain sequence is well conserved. Sequences were aligned using MAFFT and trimmed with Trimal. The phylogenetic tree was inferred using IQ-TREE with 100 bootstrap replicates. Numbers represent the bootstrap values.

S6Ks were originally identified in animal models in 1988 for their ability to phosphorylate the ribosomal protein S6 (rpS6) (Jeno et al., 1988; Nemenoff et al., 1988). It was not until 1994 that the plant S6Ks orthologues were identified in Arabidopsis (Zhang et al., 1994a; Zhang et al., 1994b). In an attempt to identify conserved AGC kinases in plants, Zhang et al. (1994b) used two degenerate oligonucleotides coding for the amino acid sequence of a conserved motif in the catalytic domains of the human protein kinase C and protein kinase A to amplify kinase homologues from Arabidopsis genomic DNA. Two genes were isolated, *Ats6k1* and *Ats6k2* (also called *atpk1/atpk6* and *atpk2/atpk19*) (Zhang et al., 1994b). The head-to-tail tandem array organization on chromosome three and the conservation of intron–exon boundaries within the genomic sequences strongly suggest that the two genes were originated by an event of gene duplication (Figure 1) (Zhang et al., 1994b; Turck et al., 1998). Initial sequence comparison studies (42.29–53.12% sequence identity with human PKA, PKCα, and S6K1 catalytic kinase domains) failed to assign AtS6K1/2 to an AGC subfamily group. In a back-to-back paper, Zhang et al. (1994a) carried out a functional characterization of AtS6K1 and identified several ribosomal proteins as potential substrates. The first *in vitro* evidence that AtS6K1/2 are rpS6 kinases came from the ectopic expression of AtS6K2 in human 293 cells and incubation of AtS6K2 isolated from plant cells with the 40 S subunit, which in both cases resulted in an increased phosphorylation of rpS6 (Turck et al., 1998). Despite the high sequence identity of AtS6K1 and AtS6K2 (88.07% overall and 96.48% for the catalytic kinase domain), an increasing number of studies have indicated that these two kinases function differently. For example, the mRNAs for *AtS6K1* and *AtS6K2* cycle with opposite phases: *AtS6K1* peaking around dawn and *AtS6K2* peaking in the afternoon (Mockler et al., 2007). Cellular localization experiments have shown that AtS6K1 is predominantly localized in the cytoplasm, while AtS6K2 resides in the nucleus (Mahfouz et al., 2006; Sun et al., 2016). However, little is known about S6K isoform-specific roles, despite initial evidence suggesting that they mediate different stress response pathways (Turck et al., 2004; Mahfouz et al., 2006; Rajamaki et al., 2017). Indeed, expression data from the Arabidopsis eFP Browser (Winter et al., 2007) show that *AtS6K1* expression is induced in response to UV-B, oxidative, and genotoxic stresses and downregulated following osmotic stress specifically in shoot, while *AtS6K2* is overexpressed in plant roots in response to salt treatment.

A comprehensive understanding of the regulation and overall biological role of plant S6Ks can only be obtained by dissecting molecular signaling pathways at the protein interaction and activity level. In this article we review expression and activation of the two S6K isoforms in plants and we discuss their roles in mediating responses to stresses and developmental cues critical for plant productivity under variable growth conditions.

## S6K STRUCTURE AND ACTIVATION

**FIGURE 2:**
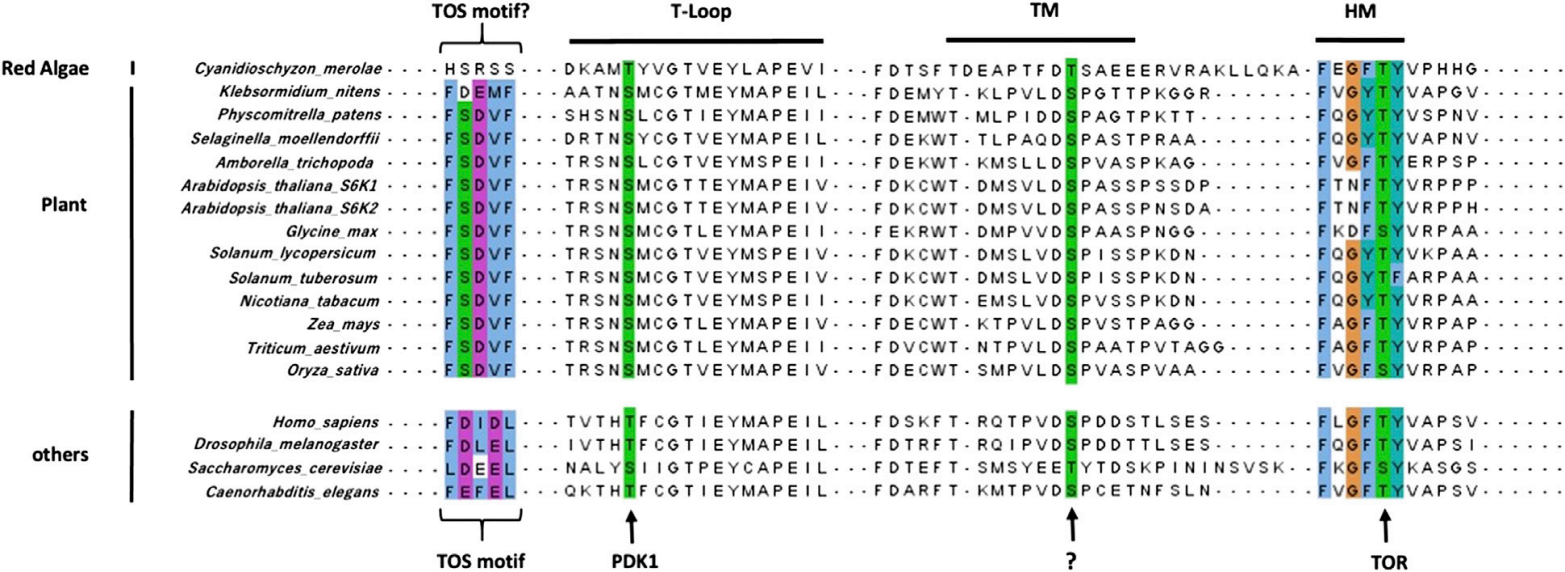
Sequence alignment of S6Ks regulatory motifs. The TOS motif, required for RAPTOR binding, is conserved in plants, albeit being different from canonical TOS motif found in other eukaryotes. The positions of the key Serine and Threonine residues phosphorylated by PDK1, TOR, and an unknown kinase in the activation loop, HM and TM respectively, are highly conserved in all S6Ks. Alignment was done using ClaustalW in the MAFFT tool on the EBI server (https://www.ebi.ac.uk/Tools/msa/mafft/) and visualized using JalView.

**FIGURE 3:**
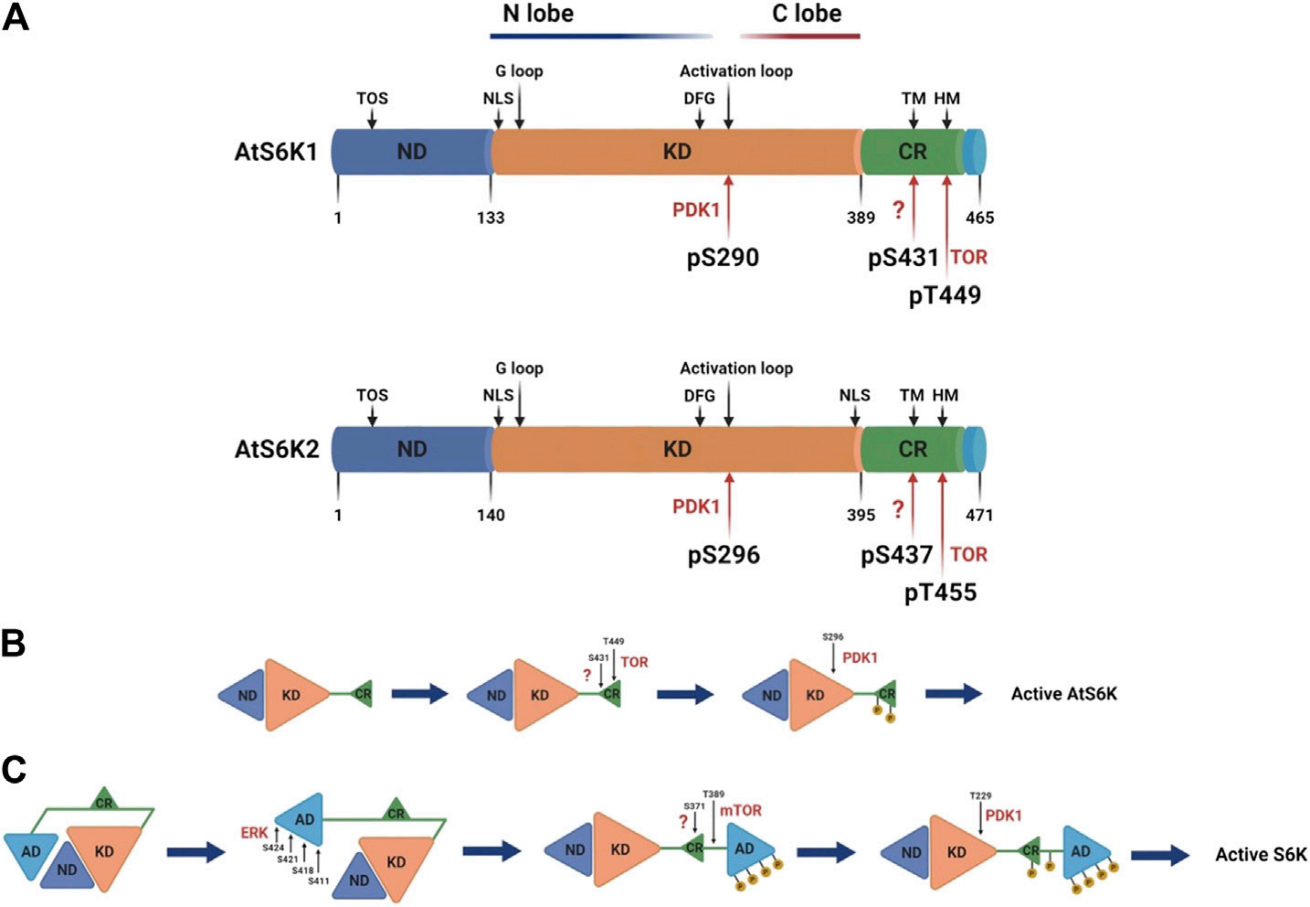
Schematic representation of AtS6Ks architecture and activation. **(A)** Comparison of AtA6K1 and AtS6K2 primary structures colored by domain. The nonconserved N-terminal domain (ND) is depicted in blue; the AGC kinase domain in orange (KD); the AGC C-terminal region in green (CR); and the nonconserved C-terminal domain in light blue. Motifs and phosphorylated residues are highlighted in the structure with black and red arrows respectively. TOS, TOR signaling motif; TM, turn motif; HM, hydrophobic motif; NLS, nuclear localization sequence; G loop, glycine rich loop. **(B)** Stepwise models of activation of Arabidopsis and **(C)** human S6Ks. The residue numbers correspond to Arabidopsis and human S6K1. AD, human S6Ks autoinhibitory domain, missing in the plant proteins, created with BioRender.com.

The Arabidopsis p70 ribosomal protein S6 kinases are a serine/threonine protein kinase family that belongs to the AGC kinases’ group (Zhang et al., 1994b). The AtS6Ks possess i) a nonconserved N-terminal regulatory domain, containing the TOR signaling motif (TOS); ii) a catalytic kinase domain with the activation loop; and (iii) a regulatory AGC C-terminal region, containing the hydrophobic motif (HM) and the Turn Motif (TM) (Turck et al., 1998; Bogre et al., 2003) (Figure 2). Although the 3D structures of AtS6K1 or 2 have not been solved and to date no plant AGC kinase domain structure is available, general kinase fold elements can be easily identified by sequence analysis (Endicott et al., 2012). AtS6K1/2 kinase domains are approximately 256 residues long, which contain an N-lobe, mainly β-sheets with only one α-helix (called C-helix), a C-lobe, predominantly α-helical, and the ATP binding site sandwiched between the two lobes (Endicott et al., 2012; Rademacher and Offringa, 2012). The C-lobe contains the activation segment (DFG-X_12_-SMCGTTEYMAPE) composed of the DFG motif, required for binding with the Mg^2+^ that orient the ATP for ɣ-phosphate transfer, the activation loop (SMCGTTEY) and the APE motif, which mediates binding of the substrates (Endicott et al., 2012; Rademacher and Offringa, 2012) (Figure 3A). The binding of ATP is also stabilized by interaction with the conserved glycine-rich loop (GXGXXG) within the N-lobe (Endicott et al., 2012; Rademacher and Offringa, 2012) (Figure 3A). Sequence analyses of AtS6Ks revealed two putative NLS (Nuclear Localization Sequence) motifs located at both ends of the kinase domain in AtS6K2, as opposed to a single NLS in AtS6K1, situated in the N–terminus of its kinase domain (Mahfouz et al., 2006) (Figure 3A). These results are in agreement with subcellular localization data, in which expression of AtS6Ks-GFP in BY2 (*Nicotiana tabacum*) cells showed that AtS6K2 is mostly confined within the nucleus, while AtS6K1 is predominantly localized in the cytoplasm, highlighting the functional diversity of the two S6K isoforms in plants (Mahfouz et al., 2006; Henriques et al., 2010).

The activity of plant S6Ks can be modulated by several factors that trigger or directly affect TOR signaling, which includes auxin and other phytohormones (Turck et al., 2004; Schepetilnikov et al., 2013; Zhang et al., 2016), insulin or insulin-like growth factors (Sánchez de Jiménez et al., 1999; Garcia Flores et al., 2001; Reyes de la Cruz et al., 2004; Dinkova et al., 2007; Garrocho-Villegas et al., 2013), nutrients (Zhang et al., 2016; Van Leene et al., 2019), a range of stress factors (Williams et al., 2003; Mahfouz et al., 2006), light signals (Turkina et al., 2011; Enganti et al., 2017; Chen et al., 2018), and also several synthetic compounds designed to target the TOR-S6K pathway (Xiong and Sheen, 2012; Schepetilnikov et al., 2013; Xiong et al., 2017). The activation of AtS6Ks is initiated by the binding of Arabidopsis TOR Complex 1 (AtTORC1) to the TOS motif, which, differently from the canonical TOS motif found in other eukaryotes, is composed of a stretch of 44 amino acid with a 12 amino acid core element (Son et al., 2017). AtTORC1 is composed of three proteins with distinct roles: i) AtTOR, a large (∼250 KDa) evolutionary conserved serine/threonine protein kinase belonging to the phosphatidylinositol 3-kinase-related kinase (PIKK) family, ii) Regulatory-Associated Protein of TOR (AtRAPTOR), which influences the activity and substrate specificity of TOR, and iii) a small Lethal with Sec Thirteen 8 (AtLST8) protein, involved in TOR-mediated signaling processes (Anderson et al., 2005; Moreau et al., 2012; Rexin et al., 2015). An increasing amount of evidence has shown that TORC1 is the only TOR complex in plants, differently from mammals which have two functionally distinct TOR high molecular weight complexes, TORC1 and TORC2 (Maegawa et al., 2015). Following direct interaction between AtS6Ks and AtRAPTOR (Sun et al., 2016), AtTOR phosphorylates the T449 and T455 within the HM (**F**TN**FT**_**p**_ **YV**R**P**) located on the AGC C-terminal region of AtS6K1 and AtS6K2 respectively (Figure 3B) (Xiong and Sheen, 2012). Phosphorylation of S431 and S437 within the TM of AtS6K1 and AtS6K2, respectively, is mediated by a yet-to-be identified kinase, but this phosphorylation step seems indispensable for AtS6K1/2 activation (Phin et al., 2003; Yaguchi and Kozaki, 2018; Yaguchi et al., 2020). HM phosphorylation creates a docking site, called PDK1 interacting fragment (PIF) (Biondi et al., 2000; Frodin et al., 2002), recognized by *Arabidopsis* 3-Phosphoinositide-Dependent Kinase 1 (AtPDK1), which directly binds AtS6Ks and phosphorylates Ser-290 and Ser-296 on the activation loop (**S**_**p**_MC**GT**E**EY**) of AtS6K1 and AtS6K2 respectively (Mahfouz et al., 2006). In many AGC-kinases the phosphorylated PIF has also been shown to enhance kinase activity by folding back on to the N-lobe of the kinase domain (Kannan et al., 2007), but no studies have investigated this mechanism in plant S6Ks.

Phosphorylation of the activation loop is a well-characterized conserved priming mechanism that enables kinases to phosphorylate their substrates (Rademacher and Offringa, 2012). Specifically, in a resting state the activation loop is usually disordered, preventing the binding of both Mg^2+^ and substrates (Nolen et al., 2004). Phosphorylation of the activation loop promotes the formation of a network of H-bonds with the C-helix and the activation segment, resulting in an “open” structure conformation allowing binding and phosphorylation of a wide range of substrates (Huse and Kuriyan, 2002). Interestingly other posttranslational modifications (PTMs) have been reported in human S6Ks, but, due to limited studies on plants, no information about the presence and roles of additional PTMs on AtS6Ks is available (Wang et al., 2008; Gwalter et al., 2009; Fenton et al., 2010).

Despite functional conservation with the human S6Ks (Turck et al., 1998), the activation mechanism of plant and human proteins is significantly different. The human S6Ks are activated by ERK, which phosphorylates the S6Ks C-terminal autoinhibitory domain, which is missing in the plant proteins (Pardo and Seckl, 2013). Only upon release of this autoinhibitory segment, TORC1 and PDK1 are able to phosphorylate S6Ks (Figure 3C) (Pardo and Seckl, 2013). This would suggest that, while the kinase domain has an evolutionary conserved function (Turck et al., 1998), the N- and C-terminal of the protein may provide species-specific regulation and substrate selection (Rademacher and Offringa, 2012). The Kozaki group (Yaguchi and Kozaki, 2018; Yaguchi et al., 2020) interrogated the role of Arabidopsis, rice, maize, tomato, and soybean S6Ks phosphorylations and, although they were not able to fully characterize plants proteins activation due to shortcomings of the experimental approach adopted (i.e., the use of a heterologous system, the unquantified ectopic expression of plants proteins in *ypk3*∆ yeast cells, and the lack of protein-specific antibodies), confirmed presence of differences in plant S6Ks regulation compared to humans.

## S6K Substrates

The recognition sequence phosphorylated by S6Ks and conserved amongst the AGC kinase family is RXRXXS/T (Tavares et al., 2015). The most well-characterized substrate and primary target of S6Ks is the ribosomal protein S6 (rpS6). In Arabidopsis, two rpS6 have been identified, rp6SA and rpS6B, which are functionally equivalent (Chang et al., 2005; Creff et al., 2010). rpS6 is located in the mRNA/tRNA binding site of the 40 S subunit and is the only protein in the 40 S ribosomal subunit to be phosphorylated in a controlled way (Mahfouz et al., 2006). rpS6 phosphorylation increases cap-dependent translation in human (Roux et al., 2007), but its role in plants is largely unknown (Enganti et al., 2017). Studies on maize have shown that auxin stimulation enhanced rpS6 phosphorylation with a concomitant recruitment of 5′TOP-like mRNAs into polysomes (Levy et al., 1991; Beltran-Pena et al., 2002). This would suggest that, at least in maize tissues, rpS6 phosphorylation regulates translation of specific proteins downstream of auxin signaling (Beltran-Pena et al., 2002). In plants, rpS6 is most commonly phosphorylated on two residues S237 and S240, phosphorylations which have been detected in Arabidopsis (Enganti et al., 2017) and maize (Williams et al., 2003). The level of phosphorylation on these residues changes during the day and night. Turkina et al. (Turkina et al., 2011) carried out an extensive mass spectrometry study on Arabidopsis cytosolic rpS6 and identified a novel phosphorylation site on S231. A higher phosphorylation level was detected during the daytime and, specifically, the day/night phosphorylation ratio on S231, S237, and S240 was 2.2, 4.2, and 1.8, respectively. The authors speculated that the higher phosphorylation level of rpS6 is at least in part responsible for the higher protein synthesis during the light period (Turkina et al., 2011). The number of rpS6 phosphorylations is different in human. In fact, S6Ks phosphorylate rpS6 on S235 (structurally equivalent to S240 in Arabidopsis), S236, S240, and S244, while RSKs (absent in plants) phosphorylate only two residues (S235 and S236) (Roux et al., 2007). Despite these differences, earlier experiments by Turck et al. (Turck et al., 1998) showed that rapamycin treatment of human 293 cells induced a reduction in rpS6 phosphorylation, which was rescued by transient expression of AtS6K2. Human rpS6 phosphorylation was detected via ^32^P incorporation, so information about the residues phosphorylated by AtS6K2 is not available. However, this experiment showed that AtS6Ks are functional homologues of human S6Ks (Roux et al., 2007).

**FIGURE 4:**
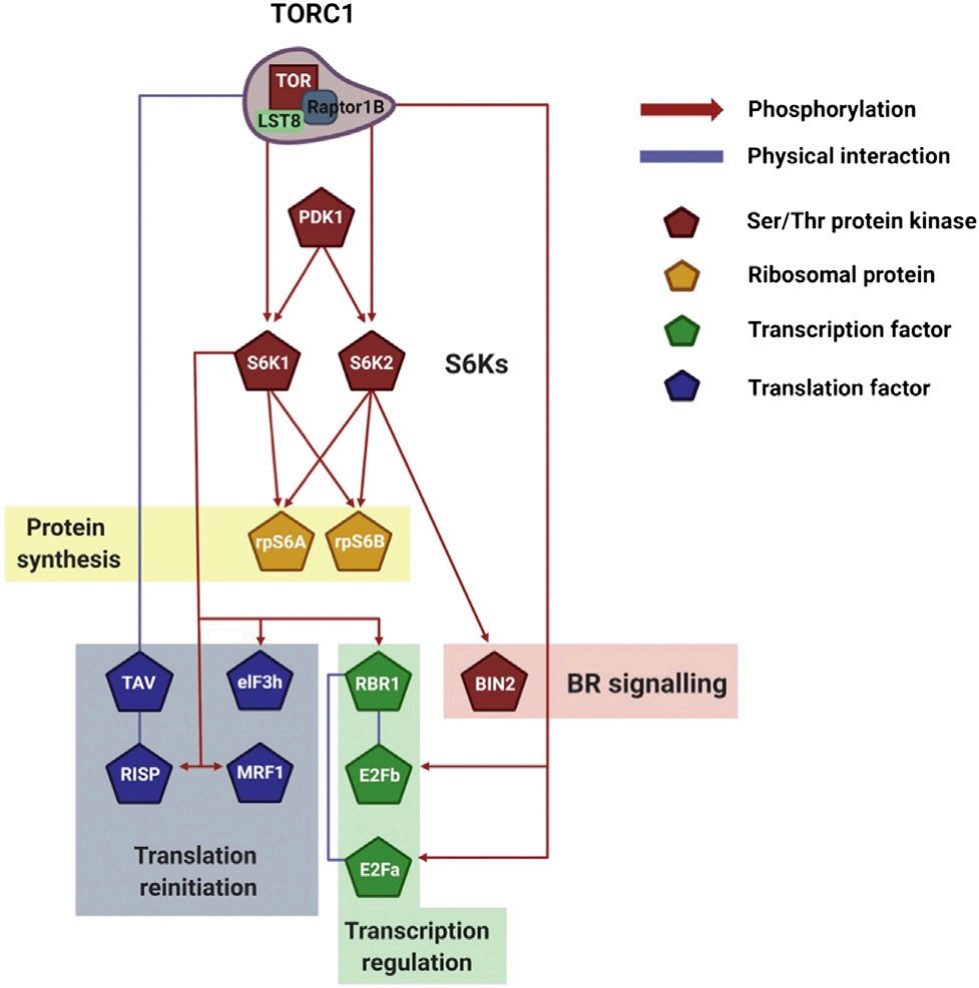
Visualization of plant S6Ks signaling network. Schematic representation of the downstream TORC1/S6Ks signaling events, which control transcription, translation, and ribosomal biogenesis. The network legend is shown on the right. Created with BioRender.com.

AtS6Ks play a major role in plant reinitiation of translation (Schepetilnikov et al., 2011; Schepetilnikov et al., 2013; Lee et al., 2017). Leet et al. (2017) recently showed that an increase in cellular energy causes phosphorylation of Arabidopsis MA3 Domain-Containing Translation Regulatory Factor 1 (MRF1) downstream of AtTOR-AtS6Ks (Figure 4). MRF is a family of translation regulatory factors composed of four isoforms, MRF1-4, which are transcriptionally and functionally regulated by TOR. Rapid phosphorylation of MRF1 under light and glucose conditions was seen to positively correlate with MRF1 association with the ribosome and eIF4A-1. In this complex, MRF1 may play a role in helping eIF4A-1 catalyze the unwinding of mRNA at the 5′-UTR to facilitate ribosome scanning (Lee et al., 2017). The authors concluded that the TOR-S6Ks-MRF1 pathway has a potential role in rapidly rebooting translation when the environment is favorable for growth (Lee et al., 2017). Similarly, association of AtS6K1 with the translation reinitiation-promoting factor eIF3 noncore subunit h (eIF3h) is triggered by the phytohormone auxin downstream of AtTOR (Schepetilnikov et al., 2013) (Figure 4). The data presented by Schepetilnikov et al. (Schepetilnikov et al., 2013) showed that in a resting state AtS6K1 is inactive and bound to polysomes. Upon auxin treatment, AtTORC1 associate with the polysome, phosphorylates AtS6K1, which in turns phosphorylates eIF3h. This activates eIF3h, which increases ribosomal loading of uORF-containing mRNAs, thus promoting, among others, translation of auxin response factors (ARFs) and basic zipper transcription factors (bZIPs) (Kim et al., 2004; Schepetilnikov et al., 2013).

An interesting case of reinitiation is induced by TAV, the Cauliflower mosaic virus (CaMV) reinitiation factor (Schepetilnikov et al., 2011). TAV binds and activates AtTOR, resulting in polysome association and AtS6K1 phosphorylation. AtS6K1 in turn phosphorylates the reinitiation-supporting protein (RISP) on S267. Importantly, TAV binds preferentially phosphorylated RISP and maintains it associated to the polysome. In this way, after termination of translation, ribosomes are equipped with the necessary machinery to reinitiate translation of the downstream ORFs (Thiebeauld et al., 2009; Schepetilnikov et al., 2011).

AtS6K1 is able to repress cell proliferation under nutrient-limiting conditions by directly binding and activating Retinoblastoma-Related 1 (AtRBR or AtRBR1; Figure 4) (Henriques et al., 2010). AtRBR is a highly conserved protein that inhibits the activity of the transcription factors E2Fs (E2FA/B/C) (van den Heuvel and Dyson, 2008). Coimmunoprecipitation experiments have shown that AtS6K1 can bind directly with AtRBR. Although experimental evidence is available only for AtS6K1, both AtS6K1 and AtS6K2 contain the LxCxE-like motif on the N-term domain required for AtRBR binding. Interestingly, E2FB was also detected in coimmunoprecipitation experiments and its relative abundance was higher in presence of AtS6K1. This could suggest that AtS6K1 is able to associate with the AtRBR-E2Fs complex. Although it is tempting to speculate that AtRBR is phosphorylated by AtS6K1 upon binding, no experimental data have linked a specific phosphorylation on AtRBR to AtS6K1. The binding of AtS6K1 results in the nuclear localization of AtRBR-E2Fs and, conversely, RNAi silencing of AtS6K1 increases the cytosol amount of AtRBR. Importantly, silencing of AtS6K1 or reduction of AtS6K1/2 (*s6k1s6k2/++* hemizygous mutants) caused an increase in chromosome numbers, which was linked to the downregulation of AtRBR and concomitant increase in E2Fs activity (Henriques et al., 2010).

Studies focusing specifically on S6K2 are scarce, in both human and plants (Pardo and Seckl, 2013). Xiong et al. (Xiong et al., 2017) showed that AtS6K2, but not AtS6K1, regulates photoautotrophic growth downstream of AtTOR. In order to map the network of AtTOR-AtS6K2 interaction, the authors used a yeast two-hybrid screening and identified Brassinosteroid Insensitive 2 (BIN2) as a key binding partner of AtS6K2 (Figure 4). BIN2, one of ten GSK3-like kinases in Arabidopsis, is responsible for blocking the transduction of brassinosteroids (BRs) signals by phosphorylating and deactivating the transcription factors BES1 and BZR1 (Peng et al., 2008; Li et al., 2020). Specifically, BIN2 is a homologue of human GSK3β and, since GSK3β is phosphorylated by human S6Ks, Xiong et al. (Xiong et al., 2017) carried out kinase assays and coimmunoprecipitation experiments to study if a similar regulation was also present in plants. The authors elegantly showed that BIN2 directly binds AtS6K2, but not AtS6K1, and is phosphorylated on S187 and S203 by AtS6K2 in an AtTOR-dependent manner. Taken together these data would suggest that AtTOR-AtS6K2-BIN2 pathway negatively regulates photoautotrophic growth (Xiong et al., 2017).

## S6K: Growth and Development

Plant growth is a function of a series of cell divisions and expansion which occur in specialized regions known as meristems. The meristems, shoot apical meristem (SAM) and root apical meristem, are made up of continuously dividing and growing cells which later form specialized tissues during cell expansion and differentiation. S6Ks have been linked to plant growth and development especially in meristems and regions of active cell proliferation. For example, expression of S6K1 was observed in both apical meristems and fast-growing organs like lateral root tips and reproductive tissues of Arabidopsis (Zhang et al., 1994b; Tzeng et al., 2009). In Arabidopsis protoplasts silencing of AtS6Ks led to an increase in cell number and cell cycle regulators, suggesting a negative effect of AtS6Ks on cell proliferation (Henriques et al., 2010). S6Ks involvement in regulating cell size was also highlighted when ectopic expression of Lily S6K1 (LS6K1) in Arabidopsis resulted in flowers with short petals and stamens due to a significant inhibition of cell expansion (Tzeng et al., 2009). This implies, at least in Arabidopsis, that S6Ks are involved in the transition from cell division to cell expansion. In agreement with this, disruption of both AtS6K1 and 2 led to halted embryo development and a reduction in epidermal leaf size as a result of inadequate cell expansion, rather than cell division, demonstrating S6Ks role in plant growth and development (Henriques et al., 2010).

The phosphorylation status of plant S6Ks during periods of accelerated growth also links S6Ks to growth responses. For example, phosphorylated maize S6K (ZmS6K) isolated from germinating maize axes positively correlated with germination time, suggesting that plant S6Ks activity is developmentally regulated (Reyes de la Cruz et al., 2004; Dinkova et al., 2007). In addition, S6Ks phosphorylation is equally observed when plant growth is stimulated exogenously with hormones, sugars, or growth factors, linking growth to S6Ks activity (Garcia Flores et al., 2001; Xiong and Sheen, 2012; Schepetilnikov et al., 2013; Van Leene et al., 2019). Cyclodipeptides (CDPs) produced by several bacteria species (plant-growth-promoting-rhizobacteria), known to alter plant root and shoot architecture by altering hormonal responses (Kimura et al., 2005; Ortiz-Castro et al., 2011; Gonzalez et al., 2017; Ortiz-Castro et al., 2020), have been linked with S6Ks phosphorylation. Indeed, Corona-Sánchez et al. (2019) showed that the increase in plant growth by *Pseudomonas aeruginosa* CDPs promoted S6K phosphorylation and activation.

It is suggested that the phosphorylation of rpS6 by S6Ks downstream of the TOR pathway is needed to promote protein synthesis required for growth processes (Ren et al., 2012). Therefore, the phosphorylation status of rpS6 is used to monitor S6Ks activity since S6Ks are the only known proteins to phosphorylate rpS6 in plants. In fact, increased growth of hormone-stimulated maize axes was positively correlated with an enhanced rpS6 phosphorylation, which was blocked by the application of rapamycin, an inhibitor of the TOR-S6K signaling pathway (Reyes de la Cruz et al., 2004; Dinkova et al., 2007). A similar result was observed with germinating maize callus, suggesting that functional TOR-S6K activity is essential for rpS6 phosphorylation during plant growth (Garrocho-Villegas et al., 2013).

Growth inhibition also induces changes in S6K activity. For example, treatment of 10-day-old maize seedlings with high doses of oligogalacturonides (OGs), a plant growth regulator which inhibits coleoptile growth, induced changes in ZmS6K activity in a similar way to rapamycin-treated seedling (Peña-Uribe et al., 2012). Varying degrees of rpS6 phosphorylation was also observed in Arabidopsis leaves at different levels of photosynthetic capacity, suggesting that S6Ks could be modulated by photosynthesis via TOR (Boex-Fontvieille et al., 2013). Indeed, disrupted mutants of rice S6K1 (OsS6K1) displayed yellow-green leaves and defective thylakoid grana due to a reduction in transcripts of genes involved in thylakoid membrane galactolipid biosynthesis. As a consequence, photosynthesis is reduced, limiting the carbon source important for plant growth, ultimately resulting in reduced growth (Sun et al., 2016). This produced a phenotype akin to that induced by TOR-inactivated mutants, which was shown to cause downregulation of nuclear genes which code for many chloroplast proteins (Dobrenel et al., 2016b; Xiong et al., 2017). Owing to the TOR pathway being the central hub for integrating environmental signals to regulate plant growth and development (Deprost et al., 2007; Dobrenel et al., 2016a; Zhang et al., 2016; Ryabova et al., 2019), loss of function mutations of members of the TORC1 complex have also been reported to have varying developmental phenotypes in plants (Menand et al., 2002; Anderson et al., 2005; Deprost et al., 2005; Moreau et al., 2012). The same is true downstream of TORC1 with S6Ks and rpS6 genetic mutants exhibiting varying developmental phenotypes, including smaller leaves, increased trichome branching, decreased root growth, and arrested embryo development (Henriques et al., 2010; Kim et al., 2014; Xiong et al., 2017). This clearly demonstrates that modulation of the TOR-S6K pathway and its associated components is crucial for plant growth and development, especially in unfavorable conditions.

## S6K and Stress

In a fluctuating environment, the biggest challenge to plant survival is the ability to adapt to stressful conditions. The impact of stress on plant growth is well documented and involves a complex coordination of physiological processes to maintain homeostasis (Kliebenstein, 2016; Bechtold et al., 2018; Bechtold and Field, 2018). Factors that modulate the TOR pathway in turn impact the activity of S6Ks thereby affecting growth. One such factor is abiotic stress. Mahfouz et al. (2006) showed that the activity of AtS6K1 was significantly reduced under osmotic stress in a TOR-dependent manner, suggesting that the TOR-S6K1 pathway is modulated by osmotic stress. Conversely, transgenic seeds constitutively expressing AtS6K1 were hypersensitive to osmotic stress (Mahfouz et al., 2006), suggesting that plants cope with stressful situations by actively reducing S6K activity via the TOR pathway, thereby limiting growth. In support of this hypothesis, a significant drop in mitotic index, a measure of cell proliferation, during heat stress was accompanied by a complete dephosphorylation of rpS6 in tomato cell cultures (Scharf and Nover, 1982). Considering growth arrest being one of the major consequences of heat stress in plants (Mittler et al., 2012; Vile et al., 2012; Bita and Gerats, 2013; Albihlal et al., 2018), the increase in mitotic index prior to rpS6 rephosphorylation was observed during recovery, implicating S6K in stress-related growth arrest (Scharf and Nover, 1982). Although S6K regulation during heat stress was relatively unknown at the time, its involvement became prominent when Turck et al. (Turck et al., 1998) showed that AtS6K2 was unable to phosphorylate mammalian rpS6 at high temperatures, due to kinase inactivation*.*

Other types of stresses which affect growth have also been linked with S6Ks activity, mostly by measuring the phosphorylation status of rpS6. Williams et al. (2003) reported a reduction in rpS6 phosphorylation in response to oxygen deprivation and a subsequent increase following reoxygenation in maize root tips. A similar dephosphorylation of rpS6 was also observed in response to heat stress (Williams et al., 2003; Enganti et al., 2017). In contrast, cold stress stimulated an accumulation of phosphorylated rpS6 (Williams et al., 2003; Enganti et al., 2017), while salt stress had no significant effect (Williams et al., 2003). Although these experiments do not provide direct evidence of endogenous S6Ks activity under stress conditions, due to protein levels falling below the detection limits of western blots (Turck et al., 1998; Turck et al., 2004), there is a positive correlation (Meyuhas, 2015).

## Conclusion and Future Directions

The phosphorylation of the ribosome was first described in 1970 (Loeb and Blat, 1970), with the first ribosomal kinase (S6K1) identified 30 and 20 years ago in humans (Krieg et al., 1988) and plants (Zhang et al., 1994a; Zhang et al., 1994b), respectively. Over the last two decades we have seen an exponential increase in publications focusing on S6Ks, due to the strong connection between this kinase family and human diseases (Tavares et al., 2015). However, our understanding of the biological roles of the S6K family in plants is limited, despite strong evidence suggesting that S6Ks regulate responses to stresses and developmental cues (Table 1) (Turck et al., 2004; Mahfouz et al., 2006; Rajamaki et al., 2017). For example, very few studies have investigated the expression at the transcriptional level of plant S6Ks in response to abiotic stresses. To date, information about changes in *AtS6K* mRNA levels is available only for cold and salinity stresses (Mizoguchi et al., 1995). However, initial analysis of data from public transcriptomic repositories suggests that both *AtS6Ks* are expressed in response to other stresses, such as drought, hypoxia, UV-B, genotoxic, oxidative, and osmotic stress (Winter et al., 2007; Hruz et al., 2008). Furthermore, whether or not this stress-induced accumulation of S6Ks transcripts corresponds to an increase in proteins amounts and kinases activity *in vivo* is still largely unknown. The complex and diverse roles of the S6K isoforms in plants adaptations to these external challenges warrant further investigation.

**TABLE 1:** Summary of AtS6K1 and AtS6k2 cellular functions. Each AtS6K1/2 cellular function has been linked to a specific AtS6K1/2 substrate or to the increased AtS6K1/2 activity, downstream of a specific signal or modification. “X” indicates that the protein target is activated by AtS6Ks; “N” indicates that the protein target is not activated by either AtS6K1 or AtS6K2; “?” indicates no information available.

Substrates	AtS6K1	AtS6K2	Cellular function and effects	Environmental trigger
rpS6A/B	X	X	Increased translation and/or 5′TOP-like dependent translation	
MRF1	X	X	Reinitiation of translation	Environment favorable for growth
RISP	X	?	Reinitiation of translation	CaMV
elF3h	X	?	Increased uORF-dependent translation	Auxin
AtRBR	X	?	Repression of cell proliferation	Nutrient-limiting conditions
BIN2	N	X	Negatively regulate photoautotrophic growth	Light, sugar, nutrients, and growth factors
?	Reduced activity	?	Stress-related growth arrest	Osmotic stress
?	?	Reduced activity	Stress-related growth arrest	High temperature
**Substrates**	**S6K1**	**S6K2**	**Mutant phenotypes**	**Mutation**
?	Reduced AtS6K1 activity	Reduced AtS6K1 activity	Halted embryo development and reduction of cell expansion	AtTORC1 and AtS6Ks
?	Reduced OsS6K1 activity	?	Impaired thylakoid function and reduced photosynthesis	OsS6K1
